# Nonfunctioning Pituitary Adenoma That Changed to a Functional Gonadotropinoma

**DOI:** 10.1155/2018/5027859

**Published:** 2018-04-29

**Authors:** Gerson Geovany Andino-Ríos, Lesly Portocarrero-Ortiz, Carlos Rojas-Guerrero, Alejandro Terrones-Lozano, Alma Ortiz-Plata, Alfredo Adolfo Reza-Albarrán

**Affiliations:** ^1^Neuroendocrinology Department, Instituto Nacional de Neurología y Neurocirugía Manuel Velasco Suárez, Ciudad de México, Mexico; ^2^Experimental Neuropathology Laboratory, Instituto Nacional de Neurología y Neurocirugía Manuel Velasco Suárez, Ciudad de México, Mexico; ^3^Endocrinology and Metabolism Department, Instituto Nacional de Ciencias Médicas y Nutrición Salvador Zubirán, Ciudad de México, Mexico

## Abstract

**Objective:**

Pituitary adenomas can be classified as clinically functional or silent. Depending on the reviewed literature, these are the first or second place in frequency of the total pituitary adenomas. Even rarer is the presence of a functional gonadotropinoma since only very few case reports exist to date. The conversion of a clinically silent to functional pituitary adenoma is extraordinarily rare; the mechanisms that explain these phenomena are unknown or not fully understood.

**Methods:**

We report the case of a woman who initially had a nonfunctional gonadotropinoma and in the course of her medical condition showed biochemical changes in her hormonal pituitary profile compatible with a functional gonadotropinoma.

**Results:**

We considered that the patient had a functional gonadotropinoma due to the hyperestrogenemia in the context of secondary amenorrhea, resolving the hyperestrogenemia after almost complete resection of the tumor.

**Conclusion:**

It is necessary to point out from a clinical and/or biochemical point of view the change in functionality that a nonfunctional pituitary adenoma may have. In the case of our patient, the suspicion of this change in functionality became evident when we found an increase in the FSH/LH ratio and a progressive increase in serum estradiol concentrations when the patient had amenorrhea.

## 1. Introduction

Nonfunctioning gonadotropinomas are the second most common type of pituitary adenoma. Its clinical diagnosis is based on the presence of symptoms associated with compression by mass effect; the true prevalence of functioning gonadotropinomas is unknown since the vast majority of reports mentioning this pituitary entity are case reports. To date, there are very few cases reported in the medical literature discussing the conversion of a nonfunctional to functional pituitary adenoma. We describe the case of a patient who initially was diagnosed with a nonfunctional gonadotropinoma that, at a later clinical follow-up, diagnosis was changed to a functional gonadotropinoma, where the main diagnostic key was the elevation of estradiol in the context of amenorrhea.

## 2. Case Report

**Figure 1:**
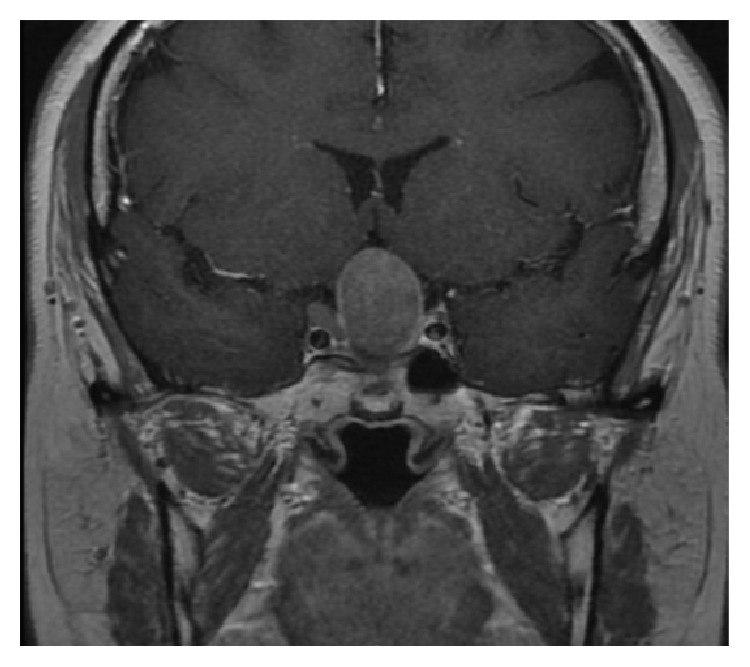
Coronal MRI T1 sequence with gadolinium. Intra- and suprasellar tumor with extension to the floor of third ventricle, after first surgery.

**Figure 2:**
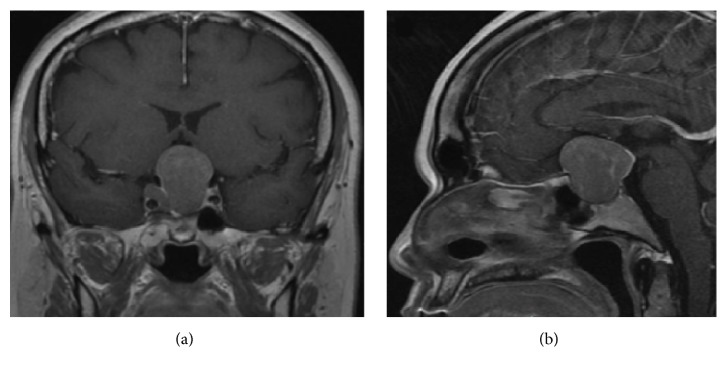
Coronal and sagittal MRI T1 sequence with gadolinium. Intra- and suprasellar tumor with tumor growth related to previous study.

**Figure 3:**
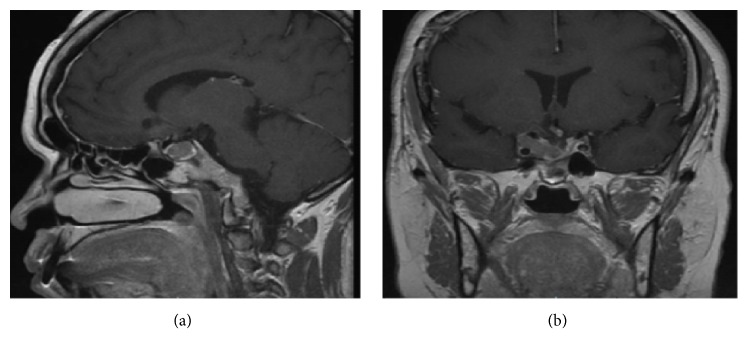
Sagittal and coronal MRI T1 sequences with gadolinium. Residual tumoral tissue only located intrasellar and right parasellar after second surgery.

**Figure 4:**
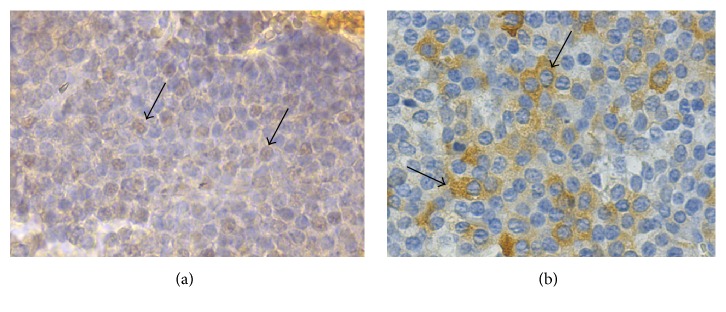
Micrography of immunohistochemistry staining with Streptavidina-Biotine technique revealed with diaminobenzidine. (a) Nuclear expression of SF-1 in the first surgery ((a), arrows). (b) Intense positivity for the FSH hormone in the second surgery ((b), arrows) (×400).

A 41-year-old woman came in for consultation in July 2013. Her menarche was at age of 13, no pregnancies occurred, and her menstrual cycles were regular, every 28–30 days. At age of 36 she had noticed oligomenorrhea and subsequently amenorrhea. She also mentioned headache and blurred vision, so she was submitted to magnetic resonance imaging (MRI), which showed a pituitary adenoma of 43 × 40 × 29 mm. Her visual field test showed bilateral temporal hemianopia and she was then sent to a reference center. The first hormonal pituitary profile showed the following results: TSH: 1.9 *μ*IU/mL (0.34–5.60), FT_4_: 7.2 pmol/L (8.11–17.25), TT_3_: 1.3 nmol/L (0.98–2.78), LH: 2.6 mIU/mL (2.4–12.6), FSH: 7.3 mIU/mL (3.5–12.5), estradiol 14.4 pg/mL (25.0–195.0), prolactin: 20.0 ng/mL (3.3–26.7), ACTH: 15.0 pg/mL (4.7–48.8), cortisol: 12.4 *μ*g/dL (8.7–22.4), and IGF-1: 53.8 ng/mL (56–194). The patient was considered to have hypopituitarism; however, only 50 mcg oral levothyroxine treatment was started every day due to central hypothyroidism. Dynamic test was not performed to rule out secondary adrenal insufficiency due to the risk of causing pituitary apoplexy. The patient underwent transsphenoidal resection in August 2013 (Figure 1). Despite the important tumor remnant, it was not specified whether the macroscopic appearance of the tumor had any special feature that made resection difficult during surgery. Immunostaining was positive for FSH and LH. During clinical follow-up, she presented some improvement in her visual fields but amenorrhea persisted. In 2015 there was deterioration in the visual fields; new hormonal determinations showed FSH of 6.31 mIU/mL and LH of 2.20 mIU/mL, as well as increase in estradiol levels at 135 pg/mL; it was considered to perform a new tumor resection to protect the vision but a short-term surgical date could not be obtained. In March 2016, a new MRI with a focus on the sellar region was performed, finding tumor remnant growth (Figures 2(a) and 2(b)). No visual worsening was reported. A new gonadal profile was requested which showed estradiol of 394.5 pg/mL and dissociation between FSH and LH (6.19 mIU/mL and 1.98 mIU/mL, resp.); amenorrhea persisted. After the annual biochemical monitoring of the gonadal hormones, it was concluded that the gonadotropinoma became functional, so it was decided to surgically intervene by transcranial approach that resulted in a significant reduction of the tumor lesion (Figures 3(a) and 3(b)). The decision to perform a transcranial approach was taken by the neurosurgery team due to their experience with this type of approach and the objective of resecting as much tumor tissue as possible. Ten days after the second surgery, a new gonadal profile measurement was performed: FSH 2.81 mIU/mL, LH 0.57 mIU/mL, and estradiol 20.3 pg/mL. Immunostaining index (percentage of immune-positive cells) was slightly higher for FSH (38.9%; range 31–48%) than LH (35.1%; range 29–39%). MIB-1 (Ki-67) labeling index was 1.7%. Histologically tumors cells arranged in papillary pattern were found (Figures 4(a) and 4(b)). It is noteworthy highlighting that hormonal therapy with estrogen and/or progestagens was never initiated during the entire management of the patient. Unfortunately, pelvic ultrasound could not be performed before the second surgery when the possibility of a functional gonadotropinoma was considered. The patient is currently waiting to receive treatment with radiosurgery.

## 3. Discussion

Pituitary adenomas are considered the third most frequent intracranial neoplastic lesion (15%), after meningiomas and gliomas. Until recently, pituitary adenomas were considered relatively rare diseases; however, improvement in various imaging techniques has increased its detection [1]. The nonfunctioning pituitary adenomas represent 15–30% of these [2]. Nonfunctioning pituitary adenomas are considered the second most common pituitary adenoma, exceeded only by prolactinoma [3], although the silent clinical course of a large percentage of them could explain a large proportion that are not diagnosed. Usually, these tumors are manifested by the effect of mass compression, such as headache, blurred vision, and seizures. After the fourth decade of life, the nonfunctioning pituitary adenoma is the most frequent type of pituitary adenoma diagnosed. Histopathologically, most nonfunctioning pituitary adenomas have positive immunohistochemistry staining for FSH/LH [4]. The pathogenesis of functioning gonadotropinomas is unknown; several theories have been postulated, among which some that stand out are greater production of intact and biologically active FSH, hypersecretion of an abnormal form of FSH that inhibits GnRH secretion at the level of the hypothalamus, further inhibiting secretion of FSH and LH, inhibition of the union of the abnormal form of FSH to FSH receptors at the pituitary level, or loss of negative counter-regulation by estrogens towards gonadotrophins [5, 6]. Within the differential diagnoses, ovarian hyperstimulation syndrome could be the consequence of continuous hyperstimulation of biologically active forms of FSH on ovarian follicles which may lead to complications such as rupture of cysts and peritonitis, hypovolemic shock, acute renal failure, distress respiratory syndrome of the adult, and pulmonary thromboembolism [7–9]. Diagnosis of functioning gonadotropinoma may be suspected of having a profile of relative gonadal level of FSH/LH > 2, hyperestrogenemia in the context of amenorrhea, and MRI (magnetic resonance imaging) showing the presence of variable size multiseptated cysts in both ovaries. Until now, it is biochemically difficult to diagnose postmenopausal women with a functioning gonadotropinoma due to them showing constant elevated FSH levels and lack of ovarian follicles to stimulate with FSH [6, 7]. Different reviews and case reports conclude that the size of functioning gonadotropinomas is always greater than 1 cm [10]. In the case of men, FSH hypersecretion can cause testicular growth; however, this clinical data might not be recognized by the physician due to the slow evolution of testicular growth. It is controversial whether this “testicular hyperstimulation” is related to testosterone concentration or not, being that some authors report that high levels of testosterone produce greater trophic effects on seminiferous tubules, while others argue that these trophic effects are directly exerted by FSH [10–12]. In a similar way to women, all reports of functioning gonadotropinoma cases in men reveal the presence of tumors larger than 1 cm. Differential diagnoses in women include ovarian hyperstimulation syndrome, hyperandrogenic chronic anovulation syndrome, and ovarian neoplasia. In men, the differential diagnosis includes McCune-Albright syndrome, congenital testicular cysts, and malignant testicular lesions [13, 14]. The management of this sellar tumor is primarily surgical. To date, there is limited data on the experience obtained with pharmacological treatment (dopaminergic agonists or somatostatin receptor agonists), with very few and even no beneficial effects. Pharmacological treatment has been displaced by radiotherapy in its various modalities, being this the second-choice therapeutic modality [6].

The patient initially presented clinically with a nonfunctioning gonadotropinoma; subsequently, the elevated levels of gonadotrophins and estradiol were evident which led to a change in the diagnosis to a functional gonadotropinoma; such change in tumor functionality is very rare. It is known that pituitary adenomas can change their gene expression and hormonal secretion; however, the exact mechanisms that explain this phenomenon are not completely clear [15]. To date, the most classic and frequent change recognized by endocrinologists is the transformation in functionality from silent corticotropinoma to Cushing's disease. Literature case reports are not clear in establishing if there has been a change in the functional pattern of the tumors previously. In other words, published clinical findings do not show a change from a nonfunctional tumor to a pattern of gonadotrophin hypersecretion due to researchers only highlighting clinical or biochemical data on hypersecretion. In our case, biochemical finding led us to diagnose a functional tumor. We consider that the progressive increase of gonadotrophins and estradiol in a patient with a pituitary adenoma and amenorrhea may suggest the presence of a functional gonadotropinoma.
